# Construction of lncRNA-m6A gene-mRNA regulatory network to identify m6A-related lncRNAs associated with the progression of lung adenocarcinoma

**DOI:** 10.1186/s12890-023-02545-x

**Published:** 2023-08-03

**Authors:** Jiangzhou Zhang, Shuheng Bai, Yanli Yan, Haojing Kang, Guangzu Li, Zhaode Feng, Wen Ma, Xuan Wang, Juan Ren

**Affiliations:** 1 Department of Oncology Radiotherapy, First Affiliated Hospital of Xi’an Jiaotong University, No. 277, Yanta West Road, Yanta District, Xi’an, Shaanxi Province 710061 China; 2 Department of Oncology, The Eighth Hospital of Wuhan, No. 1241 Zhongshan Avenue, Jiang’an District, Wuhan, Hubei Province 430010 China

**Keywords:** Lung adenocarcinoma, N6-Methyladenosine, lncRNA, Prognosis markers

## Abstract

**Background:**

We evaluated the prognostic value of m6A-related long noncoding RNAs (lncRNAs) in lung adenocarcinoma (LUAD).

**Methods:**

The expression levels of lncRNAs and mRNAs in LUAD and normal adjacent tissues from The Cancer Genome Atlas dataset were analyzed using the limma package. m6A enzyme-related differentially expressed lncRNAs and mRNAs were identified and used to construct a regulatory network. Survival analysis was performed and the correlation between lncRNAs, m6A regulators, and mRNAs was analyzed; followed by functional enrichment analysis.

**Results:**

A comparison of LUAD samples and normal tissues identified numerous differentially expressed lncRNAs and mRNAs, demonstrating that a comprehensive network was established. Two lncRNAs and six mRNAs were selected as prognosis related factors including *SH3PXD2A-AS1*, *MAD2L1*, *CCNA2*, and *CDC25C*. The pathological stage and recurrence status were identified as independent clinical factors (*P* < 0.05). The expression levels of these RNAs in the different clinical groups were consistent with those in the different risk groups. The interactions of m6A proteins, two lncRNAs, and six mRNAs were predicted, and functional analysis showed that m6A target mRNAs were involved in the cell cycle, progesterone-mediated oocyte maturation, and oocyte meiosis pathways.

**Conclusions:**

These m6A target lncRNAs and mRNAs may be promising biomarkers for predicting clinical prognosis, and the lncRNA-m6A regulator-mRNA regulatory network could improve our understanding of m6A modification in LUAD progression.

## Background

Lung cancer represents a global life-threatening malignancy. The high incidence and poor survival of patients with lung cancer leads to heavy economic and healthcare burdens. Lung adenocarcinoma (LUAD) is mainly derived from mucous and glandular epithelial proliferation around the bronchi and has been identified as the predominant subtype in patients with non-small cell lung cancer in recent years [1, 2]. Modern computed tomography screening is a reliable tool for early stage detection of LUAD, however, most cases are diagnosed at an advanced stage owing to the lack of available biomarkers. Once patients have progressed to the invasive stage, the 5-year survival rate has an average of only 15% [3, 4]. Therefore, actively investigating the molecular mechanisms of LUAD and identifying potential targets are of great significance for its diagnosis and therapy.

Long non-coding RNAs (lncRNAs) are mRNA-like transcripts that are more than 200 nucleotides long. LncRNAs can be transcribed by RNA polymerase II, 5′ capped, polyadenylated, and spliced but they cannot code for proteins owing to the lack conserved open reading frames [5]. LncRNAs typically exert diverse biological functions by interacting with regulatory proteins and are often implicated as chromatin regulators, miRNA sponges, scaffolds of RNA protein complexes, and decoys for DNA-binding factors [6, 7]. The deregulation of lncRNAs is involved in the carcinogenesis and prognosis of various cancer types. Many lncRNAs have been identified as potential diagnostic and prognostic markers in specific cancers, including the oncogenic factors *HOTAIR*, *PVT1*, and *LINC00152* [8, 9].

N6-methyladenosine (m6A) is an abundant epigenetic modification of target RNAs, including mRNA, miRNAs, circRNAs, and lncRNA [10]. The process of m6A methylation in eukaryotic cells is dynamic and reversible and is mainly controlled by three types of enzymes; methyltransferases (“writers”), demethylases (“erasers”), and binding proteins (“readers”) [11]. Most studies have focused on the m6A modification of mRNA, and only a few reports have illustrated the function of m6A modification in lncRNAs. m6A modification affects the RNA-DNA complex structure and regulates lncRNA-specific DNA binding; m6A modified lncRNA can also provide binding sites for readers and induce RNA-binding protein entry [12, 13]. Recent studies have explored the potential significance of m6A modifications and lncRNA deregulation in cancer. For instance, upregulation of the reader IGF2BP3 and lncRNA *DMDRMR* was associated with poor prognosis in renal carcinoma, and their interaction stabilized CDK4 expression, promoted G1-S transition and cancer cell proliferation [14]. Furthermore, *LINC00460* directly interacted with DHX9 and “reader” IGF2BP2 to promote *HMGA1* mRNA stability, leading to colorectal cancer cell proliferation and metastasis [15]. In lung cancer, *LCAT3* serves as a novel m6A-regulated lncRNA that promotes cancer progression by binding to FUBP1, thereby activating *c-MYC* transcription [16]. However, the mechanisms underlying the crosstalk between m6A, lncRNAs, and mRNAs remain unclear.

This study aimed to identify reliable prognostic biomarkers by constructing regulatory networks. RNA-seq expression profiles from The Cancer Genome Atlas (TCGA), combined with the m6A target gene database and co-expression analysis, were used to elucidate the post-transcriptional regulatory mechanism in LUAD. The optimal lncRNAs and mRNAs correlated with prognosis were identified using the Cox regression model. The expression patterns of candidate factors were evaluated in different clinical groups and the correlation between m6A factors, lncRNAs, and target mRNAs was analyzed. Our results provide an effective prognostic biomarker and a potential therapeutic target for patients with LUAD.

## Methods

### Data resources and preprocessing

Gene expression profiles from Illumina HiSeq 2000 (San Diego, CA, USA) RNA sequencing data in the TCGA-LUAD dataset were obtained. Each gene expression value was normalized to log_2_ (FPMM + 1). The original dataset comprised 585 samples. We retained only 559 samples with corresponding prognostic information for further analysis, including 501 LUAD tumor samples and 58 normal samples. We also downloaded the gene expression profiles of the GSE75037 dataset from the NCBI GEO database, which contained 83 control and 83 LUAD tumor samples. The data in TCGA database were used as a training dataset, and the profiles in GSE75037 served as a validation dataset. According to Ensembl_ID and annotation information, we annotated the human lncRNAs and mRNAs recorded in the HUGO Gene Nomenclature Committee database.

### Screening of differentially expressed RNAs

The Limma package version 3.34.7 [17] was used to screen differentially expressed RNAs (DERs) between the tumor and control groups. A false discovery rate (FDR) less than 0.05 and |log_2_FoldChange|>1 were selected as thresholds. pheatmap version 1.0.8 [18] was used to conduct hierarchical clustering analysis by calculating the expression values of RNAs.

### Regulatory networks construction

The m6A2Target database provides information about the writers, erasers, and readers of m6A targets [19]. In this study, the m6A2Target database was used to predict the correlation between lncRNAs, mRNAs, and m6A regulators. By comparison with the DERs we retained the intersection part of genes to construct the lncRNAs-m6A factor-mRNA regulatory network using Cytoscape version 3.6.1 [20]. The correlation between the expression of lncRNAs and mRNAs was analyzed using the cor function in R to calculate the Pearson correlation coefficient (PCC); *P* < 0.05 and an absolute PCC value greater than 0.5 were set as thresholds. RNA pairs that met these criteria were selected as co-expressed pairs for further analysis. For the candidate mRNAs, DAVID version 6.8 was utilized to conduct gene ontology (GO) terms and Kyoto Encyclopedia of Genes and Genomes (KEGG, approved by Kanehisa laboratories, Kyoto, Japan) pathway enrichment analyses [21]. The terms and pathway categories with FDR values less than 0.05 were considered significantly enriched and retained.

### Identification of crucial RNAs correlated with survival outcomes

Univariate and multivariate analyses were performed to examine prognosis-related RNAs using the survival package version 2.41-1 [22]. The samples were divided into high- and low-expression groups according to the median values of lncRNA and mRNA. The Kaplan–Meier (KM) method was then used to evaluate the correlation between the selected RNAs expression and survival outcomes.

### Expression level analysis of nodes in the comprehensive network

**Fig. 1:**
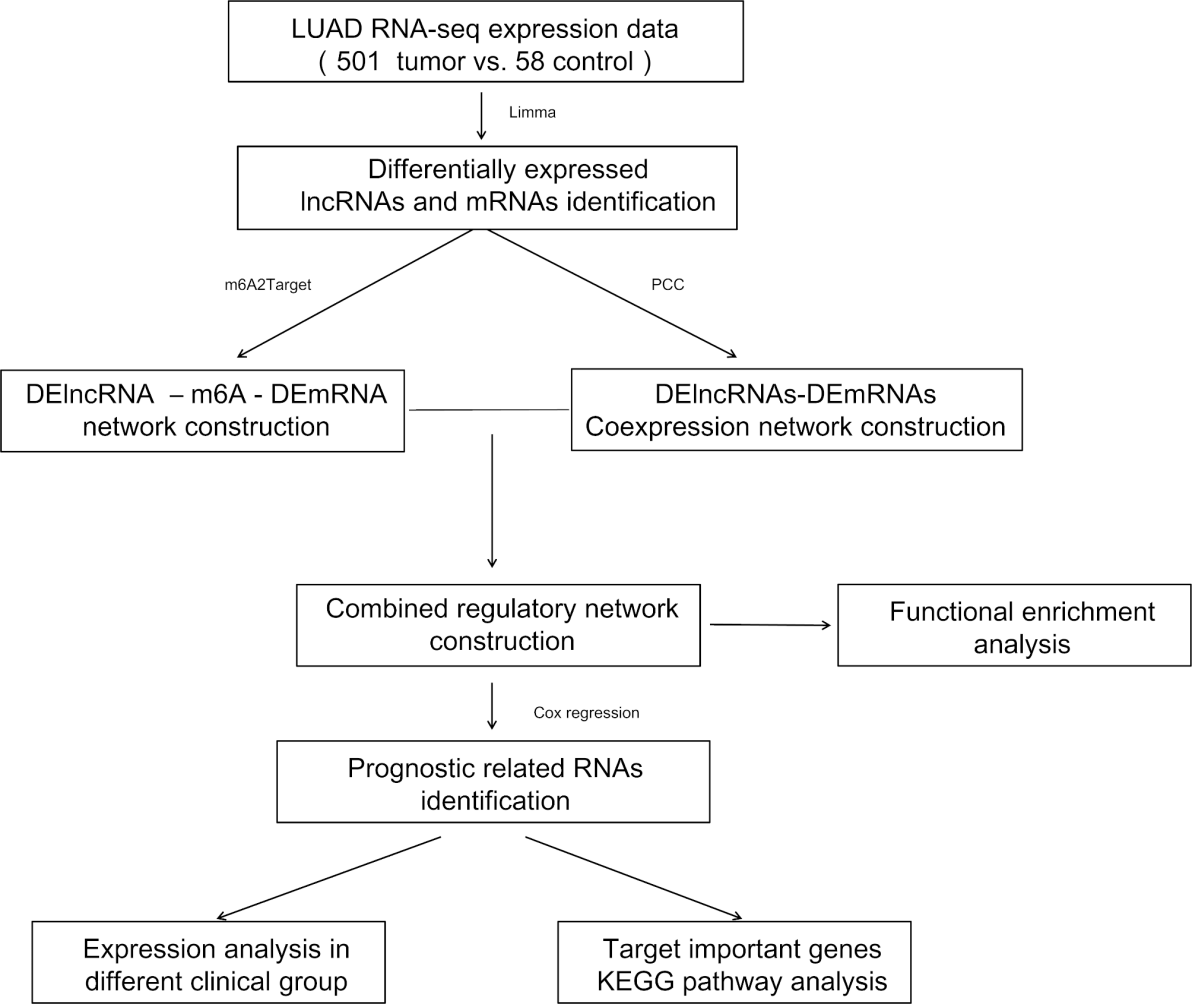
Process flow chart for the study

Combined with the clinical information of the LUAD samples in TCGA, we screened independent prognostic clinical factors using Cox regression analysis provided by the survival package version 2.41-1. A log-rank *P* value less than 0.05 was selected as a threshold. After screening for clinical factors, the tumor samples were divided into different groups, and the t-test method in R3.6.1 was used to analyze the expression levels of crucial RNAs in the different clinical groups; followed by functional analysis. A process flowchart for the study is shown in Fig. 1.

## Results

### Identification of differentially expressed RNAs associated with LUAD

**Fig. 2:**
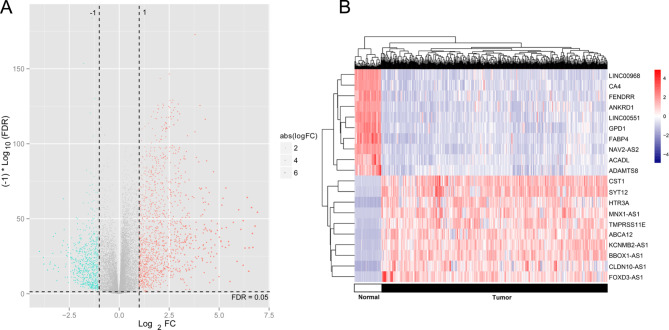
Differentially expressed RNAs (DERs) associated with lung adenocarcinoma (LUAD). (**A**). A volcano plot of the identified DERs. Red and blue dots represent significantly up and down-regulated DERs; dotted lines in horizontal and vertical directions are the criteria of FDR < 0.05 and | log_2_FoldChange | > 1, respectively. (**B**). The heat map of hierarchical clustering analysis according to expression values of top10 upregulated DERs and top10 downregulated DERs.

After removing RNAs with a median expression of 0, a total of 913 lncRNAs and 14,769 mRNAs were obtained according to the annotation information of TCGA datasets. Using the defined screening criteria, we identified 1,020 DERs between tumor and normal samples using the limma method, including 148 lncRNAs and 872 mRNAs (Fig. 2A). Hierarchical clustering showed the top10 upregulated DERs (*CST1*, *SYT12*, *HTR3A*, *MNX1-AS1*, *TMPRSS11E*, *ABCA12*, *KCNMB2-AS1*, *BBOX1-AS1*, *CLDN10-AS1*, and *FOXD3-AS1*) and top10 downregulated DERs (*LINC00968*, *CA4*, *FENDRR*, *ANKRD1*, *LINC00551*, *GPD1*, *FABP4*, *NAV2-AS2*, *ACADL*, and *ADAMTS8*) and indicated that these DERs could successfully distinguish the tumor samples from normal samples (Fig. 2B).

### Construction of lncRNA-m6A gene-mRNA network

**Fig. 3:**
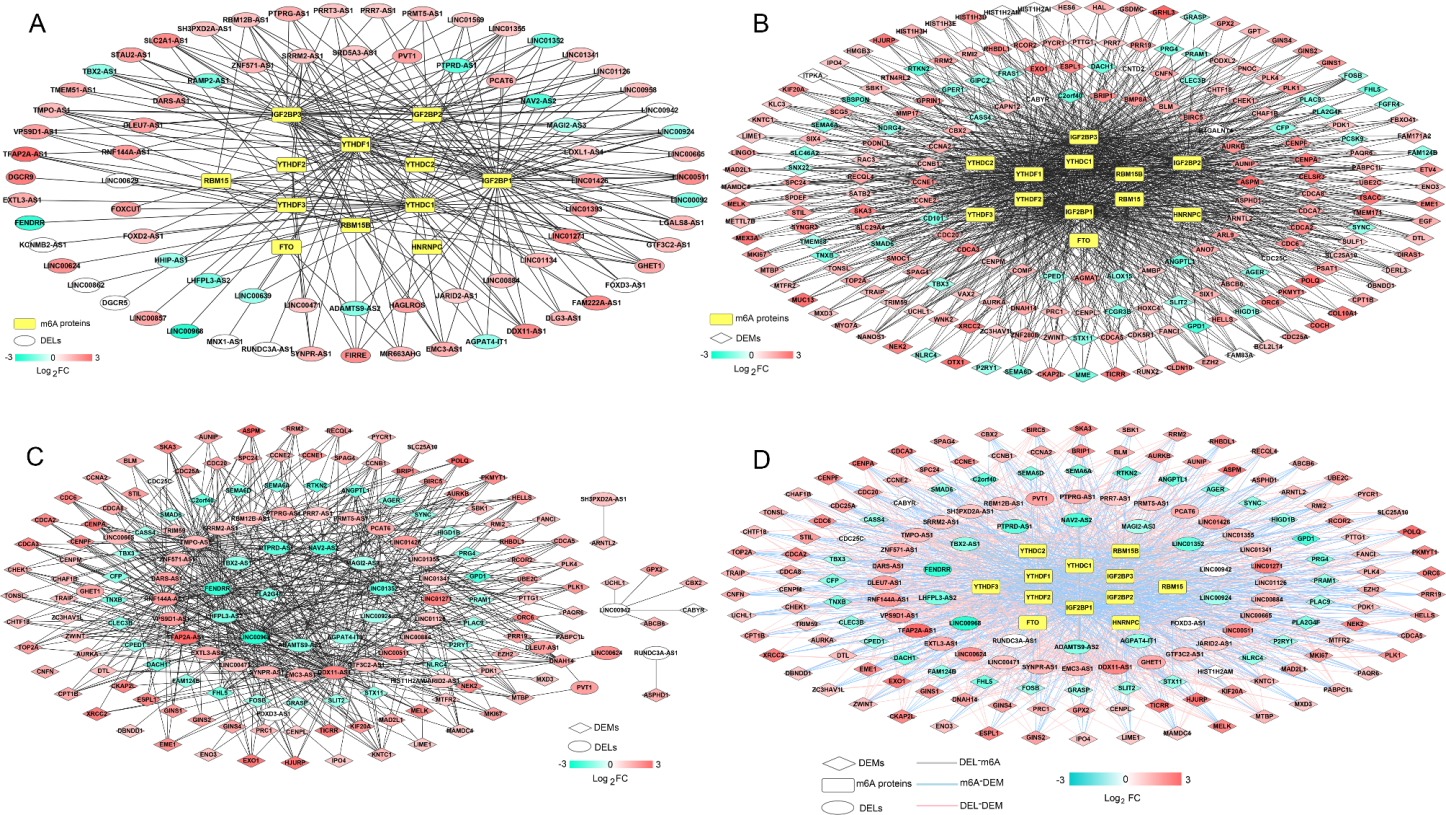
Construction of the regulatory networks. Co-expression networks showing the relationships between DELs-m6A proteins (**A**), DEMs-m6A proteins (**B**), and DELs-DEMs (**C**). DEMs: differentially expressed mRNAs; DELs: differentially expressed lncRNAs. (**D**). Comprehensive regulatory networks of DEMs, m6A proteins, and DELs. Squares, circles, and diamonds represent m6A enzymes, lncRNAs, and mRNA, respectively. Color changes from green to red indicate variations in gene expression levels from downregulation to upregulation. The gray, blue, and red lines indicate the connections between these molecules

To explore the relationship between m6A regulators and their corresponding molecules, we used the m6A2Target database to predict the potential binding between lncRNAs and mRNAs. We screened 235 RNA pairs, including 75 differentially expressed lncRNAs (DELs) and 12 m6A regulators (Fig. 3A). Similarly, candidate mRNAs linked to m6A regulators were predicted using the m6A2Target database analysis. A total of 1,055 RNA pairs associated with 206 differentially expressed mRNAs (DEMs) and 12 m6A regulators were identified by comparison with screened DEMs. The connectivity network is shown in Fig. 3B.

Interactions between DELs and DEMs were analyzed by calculating the PCC values of their expression levels. A total of 513 RNA pairs that were significantly correlated based on their expression statuses were obtained (Fig. 3C). Finally, we integrated these molecules and constructed a comprehensive regulatory network to reveal the co-relationships between lncRNA-m6A regulator-mRNAs (Fig. 3D).

**Fig. 4:**
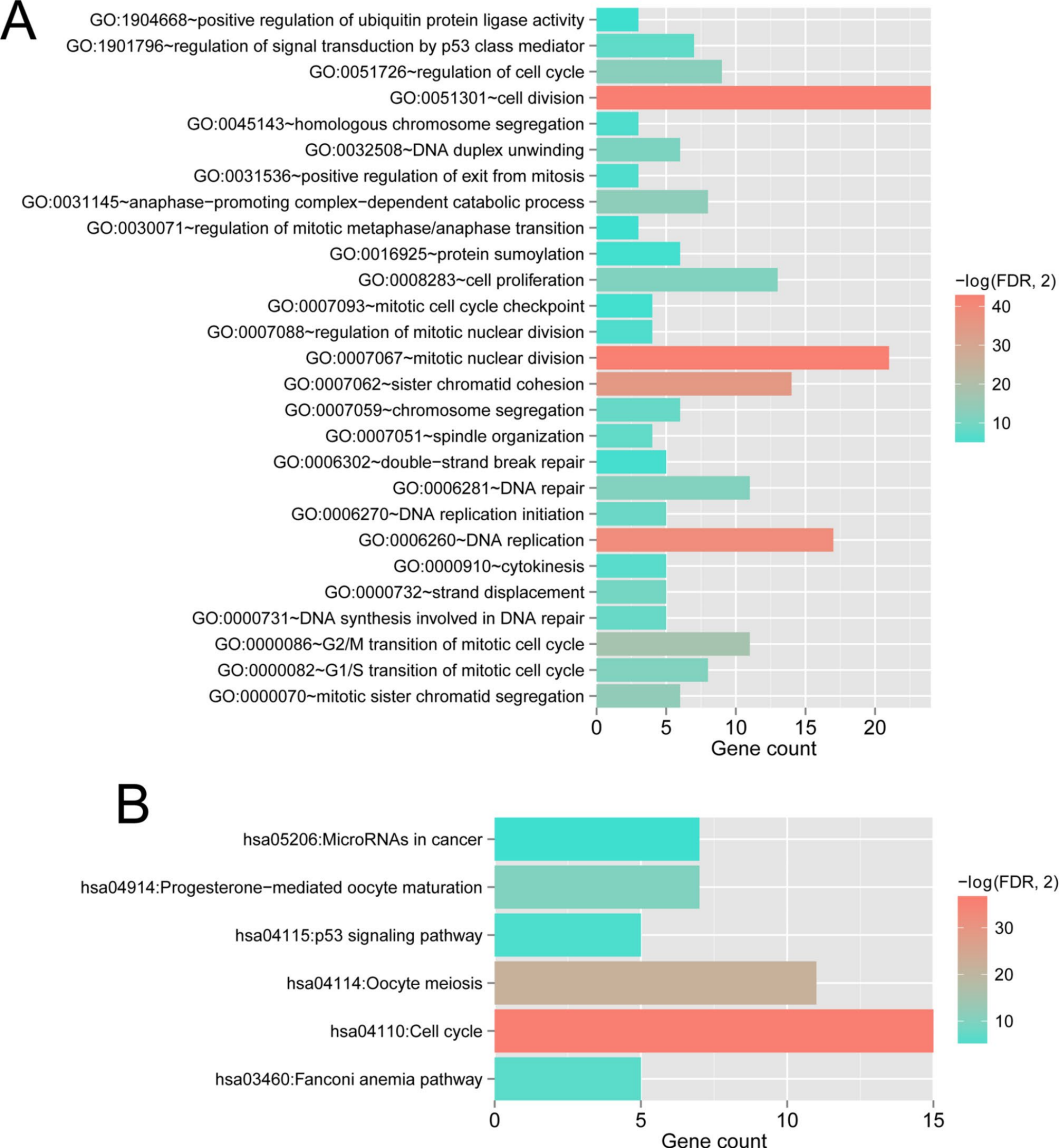
Column diagrams display the GO and KEGG (www.kegg.jp/kegg/kegg1.html, Kanehisa Laboratories, Kyoto, Japan) annotation of differentially expressed mRNAs identified from comprehensive regulatory network. Axis in horizontal and vertical direction represent the gene number and pathway categories, respectively

Functional enrichment analysis revealed that these m6A target mRNAs were associated with 27 biological processes (Fig. 4), including cell division, cell cycle regulation, mitotic nuclear division, cell proliferation, DNA replication, DNA repair, and sister chromatid cohesion. In addition, six KEGG pathways were identified: miRNAs in cancer, progesterone-mediated oocyte maturation, p53 signaling pathway, cell cycle, oocyte meiosis, and Fanconi anemia pathway.

### Identification of the crucial survival-related RNAs in LUAD and prognostic correlation analysis

Based on the nodes in the constructed comprehensive regulatory network and the clinical prognostic information of patients in TCGA, univariate Cox regression analysis was used to screen 79 RNAs, including nine lncRNAs and 70 mRNAs, that were significantly associated with prognosis. The 79 RNAs were then used for to multivariate Cox regression analysis, and eight RNAs closely related to independent prognosis in LUAD were identified, including two lncRNAs and six mRNAs.

**Fig. 5:**
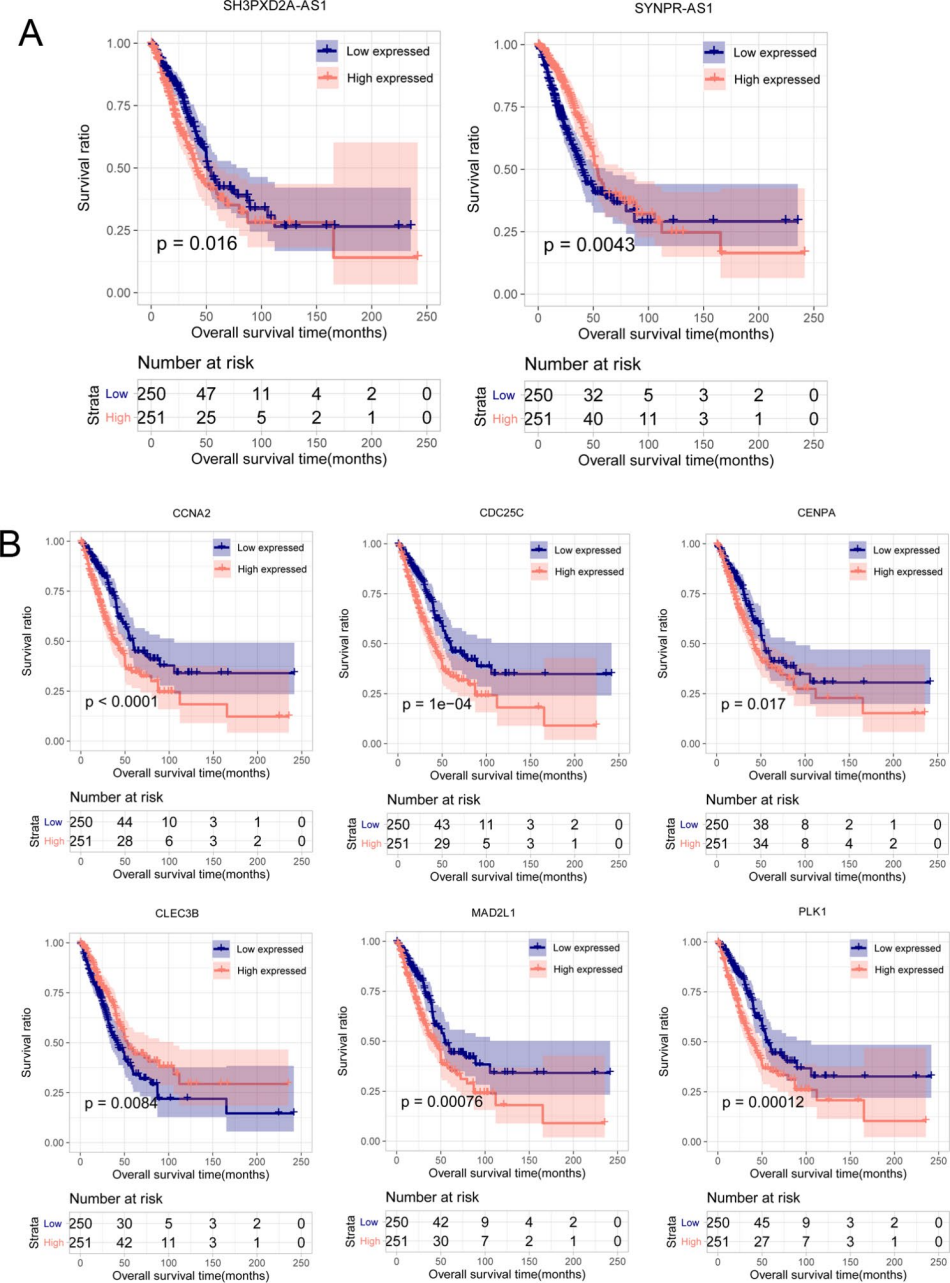
Validation of prognostic value of eight m6A regulators in TCGA database. Kaplan-Meier curves visualized the survival time of LUAD patients in high and low-expression groups for the eight RNAs. Red and blue and curves represent high and low expression groups, respectively

We then divided the LUAD samples into high- and low-expression groups, according to the median value of each regulator. Survival curves of the eight identified RNAs showed that high-expression of *SH3PXD2A-AS1*, *PLK1*, *CENPA*, *MAD2L1*, *CCNA2*, and *CDC25C* was associated with a worse overall survival time (*P* < 0.05; Fig. 5), indicating that they were oncogenic factors. High-expression of *SYNPR-AS1* and *CLEC3B* was associated with a better prognosis (*P* < 0.05; Fig. 5), suggesting that they may act as tumor suppressors.

### Expression patterns of RNAs in the different clinical groups

**Fig. 6:**
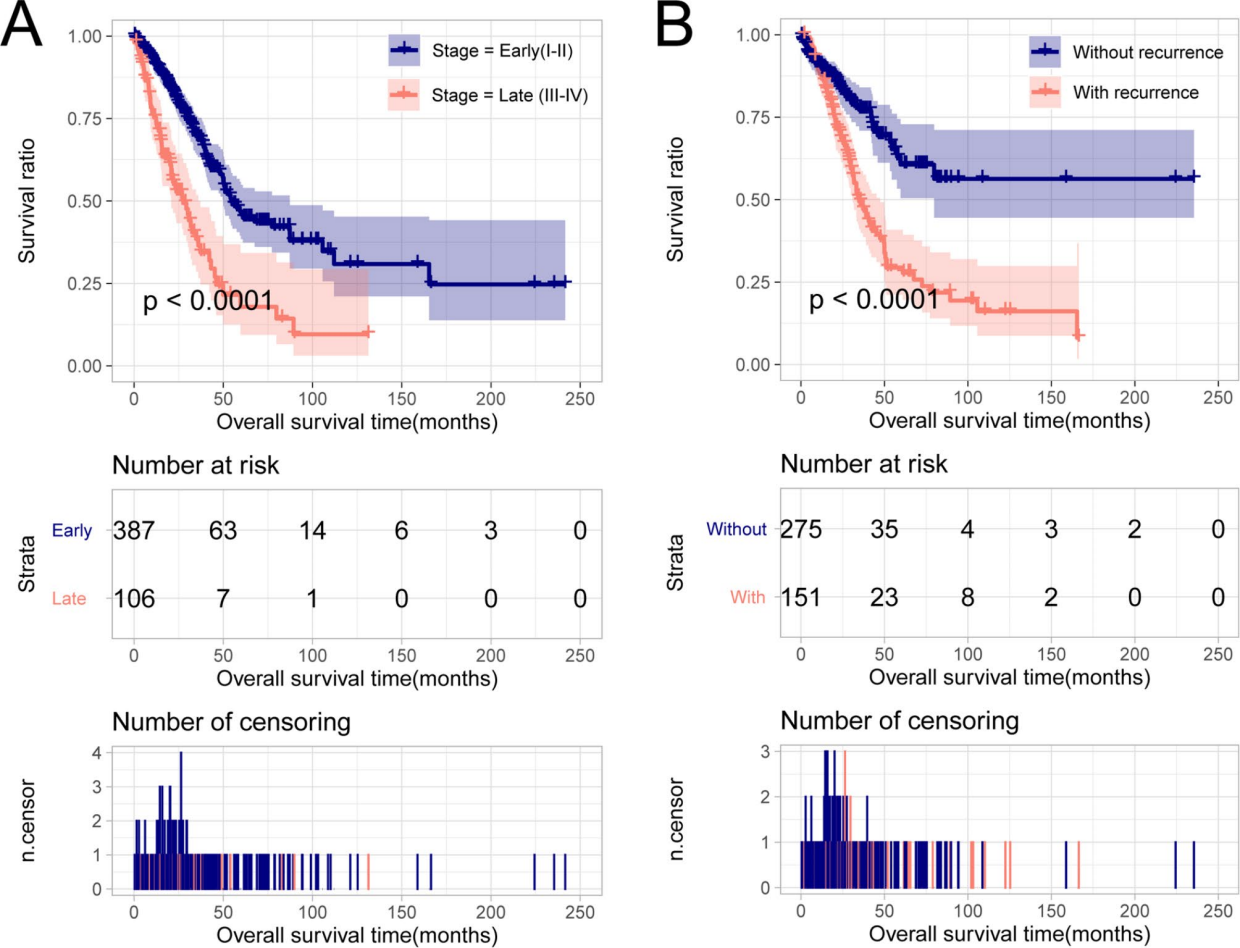
Kaplan–Meier curve of correlation between differential clinical groups and survival outcomes; early-stage vs. advanced stage (**A**) and tumor recurrence vs. without recurrence (**B**)

To evaluate the expression status of m6A target RNAs in the different clinical samples, we performed Cox regression analysis to identify independent clinical factors. Univariate analysis showed that the TNM pathological stage, pathological stage, and tumor recurrence were significantly correlated with prognosis (*P* < 0.05; Table 1). However, multivariate analysis demonstrated that only pathological stage and tumor recurrence were predicted as independent clinical factors that significantly correlated with LUAD prognosis (*P* < 0.01; Table 1). To validate the accuracy of the two clinical factors, the LUAD samples were classified into different groups according to their disease factor status. KM analysis showed that patients in the early stage (I–II) and without recurrence groups had better overall survival than those in the advanced stage (III–IV) and with recurrence groups, indicating a high accuracy for prognosis prediction (*P* < 0.001; Fig. 6).

**Table 1:** Identification independent clinical factors associated with LUAD prognosis

Clinical characteristics	Uni-variable cox		Multi-variable cox	
	HR (95% CI)	P value	HR (95% CI)	P value
Age(years, mean ± sd)	1.009[0.994–1.024]	2.64E-01	-	-
Gender(Male/Female)	1.060[0.792–1.418]	6.95E-01	-	-
Pathologic M(M0/M1)	2.111[1.232–3.616]	5.36E-03	0.236[0.0515–1.085]	6.36E-02
Pathologic N(N0/N1/N2/N3)	1.710[1.443–2.027]	2.00E-10	0.888[0.538–1.464]	6.41E-01
Pathologic T(T1/T2/T3/T4)	1.550[ 1.289–1.863]	3.10E-06	1.194[0.884–1.613]	2.48E-01
Pathologic stage(I/II/III/IV)	1.679[1.463–1.928]	2.94E-14	2.135[1.206–3.780]	9.24E-03
Tumor recurrence(Yes/No/-)	2.392[1.700-3.367]	2.44E-07	2.475[1.638–3.738]	1.68E-05
Smoking history(Yes/No)	0.765[ 0.542–1.081]	1.29E-01	-	-

**Fig. 7:**
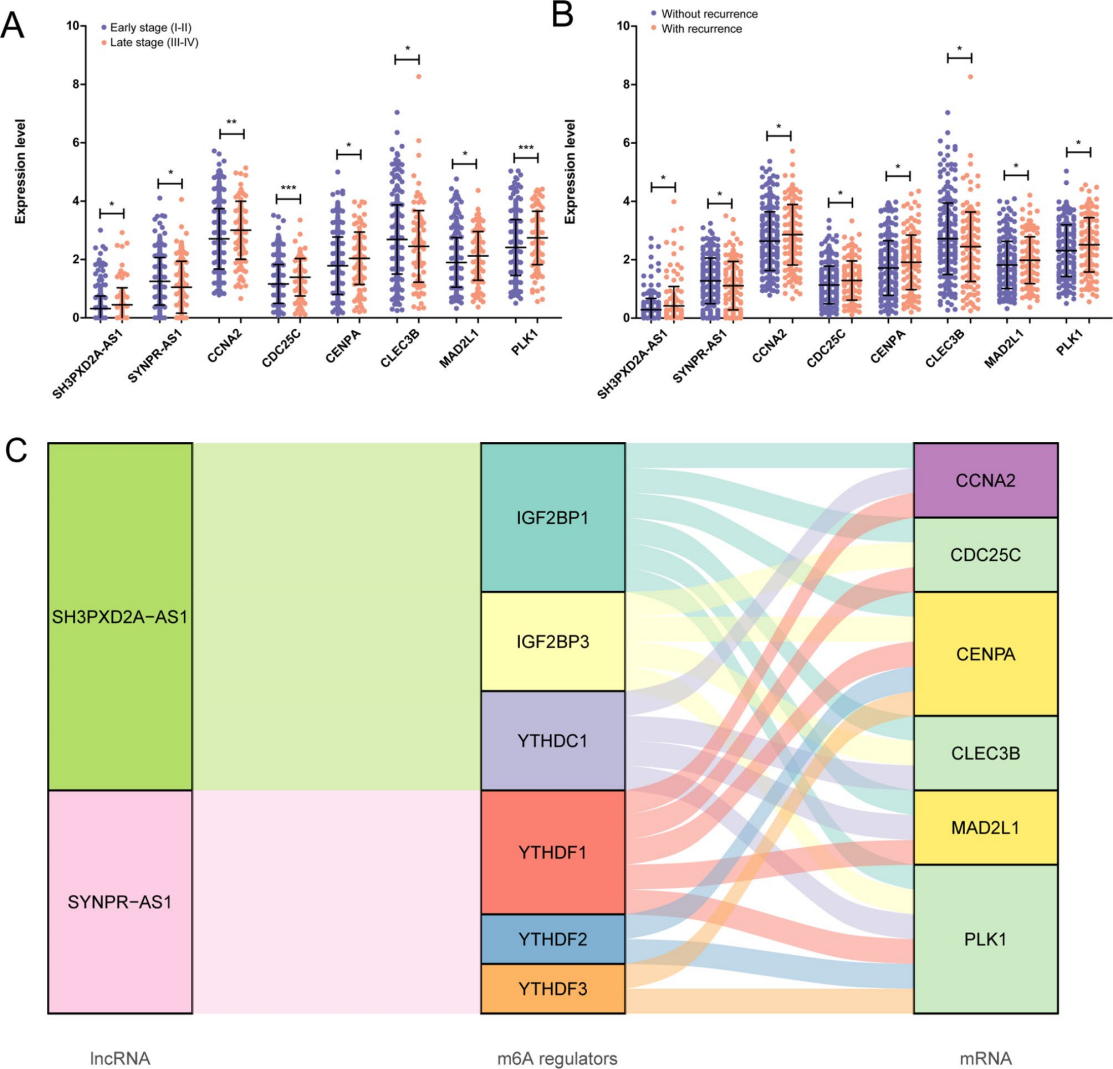
Expression pattern analysis of m6A regulators in differential clinical factor patients. (**A**) Differential expression levels of eight RNAs (two lncRNAs and six mRNAs) in various groups, such as early stage group vs. advanced pathological stage and recurrence group vs. non-recurrence group (* *P* < 0.05; ** *P* < 0.01). (**B**) Sankey diagram displaying the correlation between two lncRNAs, m6A enzymes, and six mRNAs.

Subsequently, the expression patterns of the eight RNAs were analyzed in the different pathological stage and recurrence status groups. Using the t-test method, it was found that *SH3PXD2A-AS1*, *PLK1*, *CENPA*, *MAD2L1*, *CCNA2*, and *CDC25C* were all upregulated in patients in the advanced stage and recurrence groups compared to those in the early stage and without recurrence groups, whereas *SYNPR-AS1* and *CLEC3B* displayed the opposite trend (*P* < 0.05, Fig. 7A and B). Collectively, the expression status of m6A target RNAs in the different clinical groups was consistent with that found in the different risk groups, indicating the reliable prognostic value of these molecules.

### Relationship of m6A factors, lncRNAs, and target mRNAs

We established a Sankey diagram (Fig. 7C) to determine the correlation between the six m6A enzymes and eight signatures. In the regulatory network, the lncRNA *SH3PXD2A-AS1* interacted with three m6A factors, IGF2BP1, IGF2BP3, and YTHDC1, to regulate the target mRNAs expression; whereas *SYNPR-AS1* was modulated by three other m6A factors, YTHDF1, YTHDF2, and YTHDF3.

In addition, functional analysis showed that these m6A target mRNAs were significantly enriched in 13 biological processes, including cell division, mitotic sister chromatid segregation, sister chromatid cohesion, mitotic nuclear division, viral processes, and the mitotic spindle assembly checkpoint; as well as three KEGG pathways, cell cycle, oocyte meiosis, and progesterone-mediated oocyte maturation (Table 2).

**Table 2:** Functional analysis of six m6A-reglated mRNAs.

Category	Term	Count	PValue	FDR
Biology Process	GO:0000070 ~ mitotic sister chromatid segregation	3	2.12E-05	1.68E-03
	GO:0007062 ~ sister chromatid cohesion	3	3.68E-04	1.45E-02
	GO:0007067 ~ mitotic nuclear division	3	2.11E-03	5.56E-02
	GO:0016032 ~ viral process	3	3.05E-03	6.02E-02
	GO:0051301 ~ cell division	3	4.16E-03	6.57E-02
	GO:0007094 ~ mitotic spindle assembly checkpoint	2	5.94E-03	7.82E-02
	GO:0000281 ~ mitotic cytokinesis	2	8.61E-03	9.71E-02
	GO:0000079 ~ regulation of cyclin-dependent protein serine/threonine kinase activity	2	1.16E-02	1.14E-01
	GO:0051437 ~ positive regulation of ubiquitin-protein ligase activity involved in regulation of mitotic cell cycle transition	2	2.24E-02	1.84E-01
	GO:0031145 ~ anaphase-promoting complex-dependent catabolic process	2	2.33E-02	1.84E-01
	GO:0051726 ~ regulation of cell cycle	2	3.64E-02	2.61E-01
	GO:0000086 ~ G2/M transition of mitotic cell cycle	2	4.01E-02	2.64E-01
	GO:0042787 ~ protein ubiquitination involved in ubiquitin-dependent protein catabolic process	2	4.47E-02	2.72E-01
KEGG Pathway	hsa04914: Progesterone-mediated oocyte maturation	4	1.95E-06	1.95E-05
	hsa04110: Cell cycle	4	5.72E-06	2.86E-05
	hsa04114: Oocyte meiosis	3	7.66E-04	2.55E-03

### Verification of the expression of the eight RNAs closely related to independent prognosis in the different datasets

**Fig. 8:**
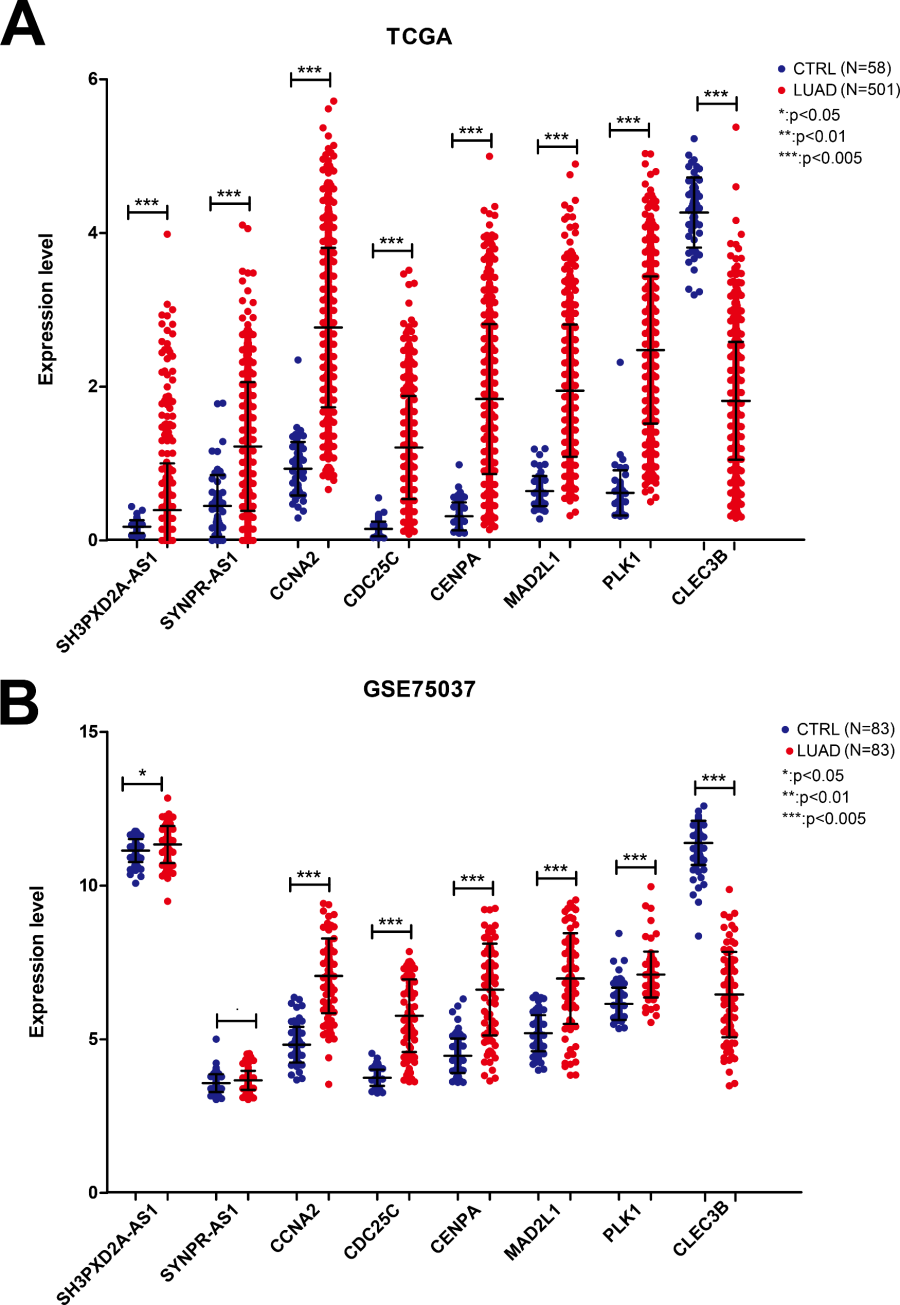
Validation of the expression of the eight RNAs, two lncRNAs and six mRNAs, in the different datasets. (**A**) Expression of the eight RNAs in the training dataset (TCGA). (**B**) Expression of the eight RNAs in the validation dataset (GSE75037).

In the training dataset (TCGA), the expression levels of *SH3PXD2A-AS1*, *SYNPR-AS1*, *CCNA2*, *CDC25C*, *CENPA*, *MAD2L1*, and *PLK1* were significantly higher in the LUAD compared to the normal samples (*P* < 0.05). However, the expression of *CLEC3B* was downregulated in LUAD compared to the normal samples (*P* < 0.05; Fig. 8A). The expression trends of *SH3PXD2A-AS1*, *SYNPR-AS1*, *CCNA2*, *CDC25C*, *CENPA*, *MAD2L1*, *PLK1*, and *CLEC3B* in the validation dataset (GSE75037) were consistent with those in the training dataset (Fig. 8B). These results indicated the relatively high reliability of the bioinformatics analysis.

## Discussion

In our study, we obtained 148 DELs and 872 DEMs in a comparison of LUAD tumor samples and normal tissues. A regulatory network was established to analyze the interactions of lncRNA-m6A mRNA. Notably, two lncRNAs and six mRNAs were found to be prognosis-related factors in LUAD. We validated the prognostic value of these novel m6A target RNAs, and the results showed that high expression of *SH3PXD2A-AS1*, *PLK1*, *CENPA*, *MAD2L1*, *CCNA2*, and *CDC25C* was associated with poor prognosis of LUADs, whereas *SYNPR-AS1* and *CLEC3B* were associated with a better prognosis (*P* < 0.05). Pathological stage and recurrence status were independent clinical factors according to the Cox regression analysis. Patients in the advanced and recurrent groups had a poor prognosis (*P* < 0.001, Fig. 6). The expression of the candidate RNAs in the advanced and recurrent groups was consistent with that in the high-risk group, suggesting the effective prognostic value of these factors. The interactions between m6A regulators and eight hub RNAs were visualized using a Sankey diagram. The m6A related mRNAs were enriched in many processes and pathway terms such as mitotic sister chromatid segregation, mitotic nuclear division, cell cycle, and oocyte meiosis.

A previous study has reported that upregulation of *SH3PXD2A-AS1* in colon cancer contributed to cancer cell proliferation, migration, and invasion [23]; it served as an endogenous sponge of *miR-330-5p* to modulate *UBA* expression. Mechanistically, *SH3PXD2A-AS1* interacted directly with p53 and regulated p53-mediated downstream gene transcription [24], however, the role of *SH3PXD2A-AS1* in LUAD remained unclear. Based on the regulatory network and survival analysis, we found that the upregulation of *SH3PXD2A-AS1* could affect the prognosis of LUAD by interacting with three m6A factors: IGF2BP1, IGF2BP3, and YTHDC1. In this study, we focused on the potential role of IGF2BP1 in the regulation of lncRNAs. IGF2BP1 and IMP-1 have been identified as conserved m6A readers that preferentially regulate m6A modified target mRNAs and enhance the stability of oncogenic factor mRNAs, leading to tumor development [25]. IGF2BP1 promotes G1/S phase transition by stabilizing E2F transcripts; a checkpoint of the cell cycle in solid cancers [26]. It controls candidate target mRNAs, *AURKA*, *HDLBP* and *YWHAZ*, mostly in a 3′ UTR-, m6A- and miRNA-dependent manner and thus enhances mRNA decay [27, 28]. A novel IMP1 inhibitor, BTYNB, disturbed the interaction between IGF2BP1 and RNAs; indicating a drug strategy for IGF2BP1-driven cancer [26, 29]. In line with previous studies, our results demonstrated the vital role of IGF2BP1 in cell cycle pathway regulation, and several candidate target mRNAs were predicted including *CCNA2* and *CDC25C*. *CCNA2* encodes the cyclin A2 protein which activates CDK1 and CDK2 to regulate the cell cycle transition [30]. Cytoplasmic localization of cyclin A2 in the S/G2 transition border triggers the activation of the mitotic kinase PLK1 [31]. *CDC25C* regulates G2/M progression and mediates DNA damage repair, and upregulation of *CDC25C* in various malignant tumors suggests that it could serve as a potential biomarker for cancer diagnosis and prognosis prediction [32]. Collectively, these findings demonstrate a broad, complex mechanism of action for IGF2BP1, *SH3PXD2A-AS1*, and their target mRNAs in promoting the cancer cell cycle.

*SYNPR-AS1* acts as a novel lncRNA and its biological role in cancer has rarely been reported. The present study found that *SYNPR-AS1* transcripts were upregulated in non-small cell lung cancer samples from TCGA [33]. However, our results showed that *SYNPR-AS1* expression was downregulated in the advanced and recurrent disease groups. This difference may be associated with the diverse genetic backgrounds and varying pathological stages of the patients. The network interaction of *SYNPR-AS1* with three m6A readers, YTHDF1, YTHDF2, and YTHDF3, was predicted. YTHDF1–3 are a class of m6A reader proteins with a conserved YTH domain. Structural analysis revealed that the m6A base fits into the YTH domain pocket via several base-specific hydrogen bonds and governs m6A-specific recognition [34]. YTHDF1 interacts with m6A marked transcripts to increase translation efficiency and protein production [35]. The relationship between lncRNAs and YTHDFs has been reported previously. YTHDF1 and YTHDF2 can read the m6A motifs and maintain the oncogenic role of the lncRNA *THOR* in an m6A-dependent manner [36]. YTHDF3 regulates YAP signaling by facilitating m6A-modified lncRNA *GAS5* degradation and mediates cancer progression [37]. In addition, upregulation of YTHDF1 is frequently found in *KRAS/TP53*-mut LUAD patients and is associated with adverse prognosis; it functionally promotes cyclin B1 mRNA translation, thereby facilitating cancer cell proliferation by regulating the cell cycle pathway [38]. In this study, correlation analysis further showed that *SYNPR-AS1* interacts with YTHDF1–3 to regulate the expression level of downstream mRNAs. Uncontrolled cell cycle signaling leads to excessive proliferation of cancer cells. The m6A target mRNAs were mainly enriched in three signaling pathways, providing promising directions for elucidating the potential mechanisms of lncRNA and mRNA signatures in LUAD. Collectively, our results demonstrate that these m6A-related lncRNAs and mRNAs may serve as reliable prognostic markers and potential targets for cancer therapy.

This study has some limitations. External validation of other LUAD datasets and more clinical samples would be beneficial to evaluate the prognostic value. Although the six m6A target mRNAs identified in this study are widely reported to be involved in cancer progression, the biological roles of the two lncRNAs have not been elucidated and their involvement in LUAD is unknown. Future experiments should be conducted to investigate the functions of lncRNAs and their association with m6A-related regulators and mRNAs.

## Conclusion

In summary, a comprehensive network of lncRNAs-m6A enzymes-target mRNAs allowed us to explore the potential functions of the candidate lncRNAs in LUAD. The systematic study of m6A modification patterns in patients with LUAD identified two lncRNAs and six mRNAs as prognostic markers. Our results provide a better understanding of the m6A regulatory mechanism in LUAD progression.

## Data Availability

The datasets generated and/or analyzed during the current study are available in the TCGA repository, https://xenabrowser.net/datapages/?dataset=TCGA-COAD.htseq_fpkm.tsv&host=https%3A%2F%2Fgdc.xenahubs.net&removeHub=https&3A%2F%2Fxena.treehouse.gi.ucsc.edu%3A443.

## Abbreviations

lncRNA: long noncoding RNAs
m6A: N 6-Methyladenosine
LUAD: lung adenocarcinoma
TCGA: The Cancer Genome Atlas
DERs: differentially expressed RNAs
GO: Gene Ontology
KEGG: Kyoto Encyclopedia of Genes and Genomes
